# Risk Factors for Diabetic Retinopathy Change With Diabetes Duration: Synergistic Effect of Long Duration and Anemia

**DOI:** 10.1155/jdr/8611325

**Published:** 2026-05-05

**Authors:** Yuan Liu, Yang Meng, Runping Duan, Baoyi Liu, Yuan Ma, Chin-Ling Tsai, Ziye Chen, Zhuojun Xu, Zitong Chen, Guanli Zhu, Tao Li

**Affiliations:** ^1^ State Key Laboratory of Ophthalmology, Zhongshan Ophthalmic Center, Sun Yat-sen University, Guangzhou, Guangdong, China, sysu.edu.cn; ^2^ Guangdong Provincial Clinical Research Center for Ocular Diseases, Zhongshan Ophthalmic Center, Sun Yat-sen University, Guangzhou, Guangdong, China, sysu.edu.cn

**Keywords:** anemia, diabetic retinopathy, disease duration, NHANES, risk factors

## Abstract

**Background/Objectives:**

The aim of this study was to identify the sociodemographic and systemic risk factors associated with diabetic retinopathy (DR) across varying durations of diabetes mellitus (DM) and to evaluate the potential effect of anemia on the occurrence of DR.

**Methods:**

The retrospective cross‐sectional study was based on the National Health and Nutrition Examination Survey (NHANES) 2005–2020, which included a total of 2,487 participants with Type 2 diabetes mellitus. Individuals were stratified into two groups based on DM duration: group 1 (≤ 10 years) and group 2 (> 10 years). Univariate weighted logistic regression and weighted multivariate logistic regression with adjustment for covariates (Models 1–3) were used to explore the risk of DR occurrence across different DM durations, as well as the synergistic effect of DM duration and anemia on DR occurrence.

**Results:**

Weighted univariate logistic regression analysis revealed that insulin use, diabetic nephropathy (DN), and education level were significantly associated with DR across both DM duration groups. In group 1, factors such as hemoglobin A1c, total cholesterol, low‐density lipoprotein cholesterol, and fasting plasma glucose were positively correlated with DR, whereas an earlier age of DM diagnosis and higher family income were protective factors. In group 2, anemia and urinary albumin‐to‐creatinine ratio were positively associated with DR, whereas eGFR was negatively associated with DR (all *p* < 0.05). A DM duration of > 10 years is an independent risk factor for DR (Model 3: *OR* = 1.53, 95% CI 1.08–2.16, *p* = 0.017). The combination of prolonged DM duration and anemia significantly increased the risk of DR, with a synergistic effect observed even after full adjustment (Model 3: *OR* = 2.24, 95% CI 1.30–3.84, *p* = 0.004).

**Conclusions:**

Sociodemographic and systemic risk factors for DR vary across DM duration groups. Insulin use, DN, and education level were significantly associated with DR in all DM duration groups. Notably, the combination of prolonged DM duration (> 10 years) and anemia significantly increased the risk of DR, demonstrating a synergistic effect.

## 1. Introduction

The prevalence of Type 2 diabetes mellitus (T2DM) is rising annually with the improvement of modern living standards and lifestyle changes [1]. Diabetic retinopathy (DR) is one of the most common microvascular complications of diabetes mellitus (DM) and the leading cause of irreversible vision loss in working‐age people worldwide [2, 3]. The presence of DR has a significant impact on patients′ quality of life, which is a major psychological and economic burden [4].

DM duration is now recognized as an important risk factor for the development of DR [4, 5]. The prevalence of DR varies specifically with the duration of DM. It has been reported that the prevalence of DR in newly diagnosed DM is 9%. The prevalence of DR in patients with DM of more than 10 years may even exceed 50% [6]. Although the duration of DM is a nonmodifiable risk factor, identifying additional sociodemographic and systemic factors that influence DR occurrence at different stages of DM can help clinicians more effectively mitigate the risk of DR in affected patients.

In addition to the well‐established role of DM duration, individuals with T2DM frequently present with anemia [7]. Previous studies indicated a correlation between anemia and the development of microvascular and macrovascular complications in individuals with T2DM [8], such as diabetic nephropathy (DN) [9], DR [10], and cardiovascular disease [11]. Qiao et al. [12] first reported the association between anemia and the development of DR, and subsequent studies have reached similar conclusions [10, 13–15]. It is speculated that the decreased hemoglobin concentration, which indicates impaired oxygen‐carrying capacity, may negatively affect the retina, increasing the risk of DR in individuals with DM [10].

The purpose of this study was to identify the sociodemographic and systemic risk factors for DR across different durations of DM and to explore the impact of anemia on DR occurrence in these patients.

## 2. Materials and Methods

### 2.1. Study Population

This was a retrospective cross‐sectional study based on the National Health and Nutrition Examination Survey (NHANES) 2005–2020. The NHANES database (https://wwwn.cdc.gov/nchs/nhanes/Search/default.aspx) contains population data, questionnaire data, laboratory data, and dietary data. It is a research program designed to assess the health and nutritional status of adults and children in the United States [16, 17]. The research protocol has been approved by the Institutional Review Board of the National Center of Health Statistics (NCHS), and all participants have provided written informed consent. As the de‐identified data analyzed in the present study were publicly available from NHANES, no further review board approval was required.

**Figure 1:**
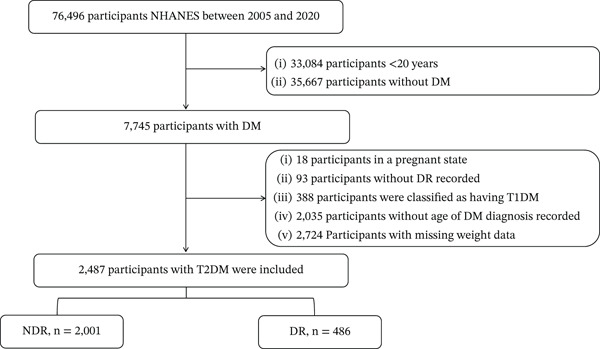
Flowchart of inclusion and exclusion criteria for the study population. NHANES, National Health and Nutrition Examination Survey; DM, diabetes mellitus; T1DM, Type 1 diabetes mellitus; T2DM, Type 2 diabetes mellitus; NDR, nondiabetic retinopathy; DR, diabetic retinopathy.

A total of 76,496 participants from 2005 to March 2020 were included in this study, whereas 33,084 participants under the age of 20 and 35,667 participants without DM or with missing DM data were excluded. We also excluded 18 participants who were pregnant, 93 participants with no DR diagnosis recorded, 388 participants diagnosed with Type 1 diabetes mellitus (T1DM), 2,035 participants without recorded age at diabetes diagnosis, and 2,724 participants with missing weight data. Ultimately, 2,487 participants with T2DM were included in the study (Figure 1).

### 2.2. Definition of T2DM and DR

DM was defined based on available NHANES questionnaire and laboratory data, using criteria established by the American Diabetes Association and previous studies. Individuals were classified as having diabetes if they met any of the following conditions: (1) a previous diagnosis of DM, (2) fasting plasma glucose (FPG) ≥ 7.0 mmol/L, (3) glycosylated hemoglobin A1c (HbA1c) ≥ 6.5%, or (4) current use of diabetes medications (diabetic pills or insulin) [18]. For participants with diagnosed diabetes, a treatment‐based algorithm was implemented to distinguish T2DM from T1DM, using self‐reported information on the type and duration of medication use. This algorithm was based on the assumption that patients with T1DM would initiate insulin treatment and not oral hypoglycemic agents within a year of diagnosis [19, 20]. The presence of DR was confirmed using data from a self‐report questionnaire (“DIQ080—Diabetes affected eyes/had retinopathy”), indicating that the respondent had been informed by a medical professional that their diabetes had affected their eyesight.

### 2.3. Definition of DM Duration

DM duration was defined as the age (in years) at screening minus the age at DM diagnosis. The age at DM diagnosis was obtained from self‐report in questionnaire data (“DID040—Age when first told you had diabetes”). Participants were categorized into two DM duration groups: ≤ 10 years (group 1) and > 10 years (group 2).

### 2.4. Definition of Anemia Status

Anemia was defined as hemoglobin levels of < 120 g/L in women and < 130 g/L in men [21].

### 2.5. Potential Confounders

We selected potential confounders based on previous research findings and clinical expertise. Race was categorized into five groups: Mexican American, other Hispanic, non‐Hispanic White, non‐Hispanic Black, and other races. Education level was classified as less than high school graduate, high school graduate or equivalent, and college graduate or above. Family income‐to‐poverty ratio was divided into three categories: < 1.5, 1.5–3.5, and ≥ 3.5.

Smoking status, based on questionnaire data, was categorized into three groups: never smoked (< 100 cigarettes in lifetime), former smoker (≥ 100 cigarettes in lifetime, but now quit), and current smoker (≥ 100 cigarettes in lifetime, still smoking) [22]. Alcohol intake was classified as nondrinkers (< 12 drinks/year) and drinkers (≥ 12 drinks/year).

Hypertension was defined as systolic blood pressure (SBP) ≥ 130 mmHg and/or diastolic blood pressure (DBP) ≥ 80 mmHg after three consecutive measurements, or a prior diagnosis of hypertension. Hyperlipidemia was defined by any of the following: total cholesterol (TC) ≥ 6.2 mmol/L, triglycerides (TG) ≥ 2.3 mmol/L, low‐density lipoproteins (LDL‐C) ≥ 4.1 mmol/L, high‐density lipoproteins (HDL‐C) < 1.0 mmol/L, or a previous diagnosis of hyperlipidemia. DN was defined by: (1) a confirmed diagnosis of diabetes and (2) a urine albumin‐to‐creatinine ratio (UACR) ≥ 30 mg/g, or estimated glomerular filtration rate (eGFR) ≤ 60 mL/min/1.73 m^2^ or both [23]. For eGFR calculation, we applied the Chronic Kidney Disease Epidemiology Collaboration (CKD‐EPI) equation [24]: eGFR CKD − EPI (mL/min/1.73 m^2^) = 141 × min (Scr/*κ*, 1)^*α*^ × max (Scr/*κ*, 1)^−1.209^ × 0.993^Age^ × 1.018 [if female] × 1.159 [if Black], where Scr is serum creatinine, *κ* is 0.7 for women and 0.9 for men, *α* is −0.329 for women and −0.411 for men, and “min” and “max” indicate the minimum and maximum values between Scr/*κ* and 1, respectively.

BMI was calculated as weight (kg) divided by height squared (m^2^). The formulas for calculating the systemic immune‐inflammation index (SII) and systemic inflammation response index (SIRI) are as follows [25]: SII = (platelet count × neutrophil count)/lymphocyte count and SIRI = (neutrophil count × monocyte count)/lymphocyte count.

Insulin use was obtained from questionnaire data and categorized as either current use (those using insulin for diabetes management) or nonuse (those not using insulin). Examination and laboratory data included SBP, DBP, waist circumference, HbA1c, TG, TC, HDL‐C, LDL‐C, and FPG.

### 2.6. Statistical Analysis

Statistical analyses were performed with R Studio Version 4.4.1 (Boston, Massachusetts, United States). Appropriate weighting methodology was employed to account for the complex sampling design, ensuring nationally representative results, as recommended by the NHANES guidelines [26]. The variables with missing values and their corresponding percentages are provided in Table S1. Missing values in the data were addressed using multiple imputation, generating five independent imputed datasets. For subsequent analyses, all statistical models were run separately on these five datasets, and the results were combined using Rubin′s rules.

Continuous variables are presented as mean ± SD and were compared across DM duration groups using weighted linear regression; categorical variables are presented as percentages (%) and were compared using the weighted chi‐square test. Weighted univariate logistic regression was employed to evaluate the association between variables and DR across various DM duration groups. Variables demonstrating statistical significance in the univariate analysis were further analyzed for correlation, with highly correlated variables excluded to address multicollinearity.

We constructed three weighted multivariate logistic regression models to assess the potential relationship between anemia status, DM duration, and the risk of DR. Model 1 adjusted for survey year, age, sex, and race; Model 2 adjusted for survey year, age, sex, race, use of insulin, educational level, DN; and Model 3 adjusted for survey year, age, sex, race, insulin use, educational level, DN, family income‐to‐poverty ratio, HbA1c, UACR, eGFR, and TC. The results were recorded as odds ratios (ORs), along with 95% confidence intervals (CIs). Statistical significance was indicated by *p* < 0.05.

## 3. Results

### 3.1. Baseline Characteristics of the Study Population

The study employed weighted analyses, which included 2,487 participants, representing a nationally weighted population of 979,300,696 individuals. The weighted mean age of the participants was 60.23 years, with 50.46% of the weighted sample being male. A total of 486 participants (16.79% weighted) had been diagnosed with DR. Group 1, consisting of individuals with DM duration ≤ 10 years, included 1,403 participants, representing 581,568,967 individuals, whereas group 2, consisting of individuals with DM duration > 10 years, included 1,084 participants, representing 397,731,728 individuals, as detailed in Figure 1 and Table 1.

**Table 1:** Baseline characteristics across different diabetes duration groups.

Characteristic	Total n = 2,487	DM duration		p value^a^
		≤ 10 years, n = 1,403	>10 years, n = 1,084	
Weighted population	979,300,696	581,568,967	397,731,728	
Age, years	60.23 (13.28)	57.17 (13.61)	64.72 (11.38)	<0.001
Sex				0.314
Male, %	50.46	49.33	52.13	
Female, %	49.54	50.67	47.87	
Age of DM diagnosis, years	49.41 (13.71)	52.65 (13.36)	44.66 (12.81)	**<0.001**
DR group				<0.001
NDR, %	83.21	87.43	77.04	
DR, %	16.79	12.57	22.96	
Anemia status				
No, %	87.14	90.47	82.28	<0.001
Yes, %	12.86	9.53	17.72	
Use of insulin				<0.001
Nonuse, %	77.10	88.48	60.46	
Current use, %	22.90	11.52	39.54	
Race				
Mexican American, *%*	9.73	10.68	8.34	0.105
Other Hispanic, %	6.42	7.05	5.51	
Non‐Hispanic White, %	59.85	57.20	63.73	
Non‐Hispanic Black, %	15.18	15.63	14.54	
Other Races ‐ Including multiracial, *%*	8.82	9.45	7.88	
Educational level				
Less than high school, *%*	25.54	22.90	29.39	0.016
High school or equivalent, %	24.26	26.39	21.13	
College or above, %	50.21	50.71	49.48	
Family income‐poverty ratio level				0.442
<1.5, %	30.21	29.23	31.64	
1.5‐3.5, %	36.39	35.94	37.04	
≥3.5, %	33.41	34.84	31.32	
Smoking status				0.111
Never (<100 cigarettes in lifetime), %	50.63	51.35	49.58	
Former (≥100 cigarettes in lifetime, but now quit), %	34.12	31.78	37.54	
Current (≥100 cigarettes in lifetime, still smoking), %	15.24	16.86	12.87	
Alcohol intake				
Nondrinkers (<12 drinks/year), %	35.37	33.16	38.59	0.057
Drinkers (≥12 drinks/year), %	64.63	66.84	61.41	
Hypertension				0.015
No, %	21.26	23.74	17.63	
Yes, %	78.74	76.26	82.37	
Hyperlipidemia				0.145
No, %	19.60	21.06	17.44	
Yes, %	80.40	78.94	82.56	
Diabetic nephropathy				<0.001
No, %	60.32	67.54	49.75	
Yes, %	39.68	32.46	50.25	
SBP, mmHg	114.03 (45.41)	113.37 (43.82)	115.01 (47.65)	0.561
DBP, mmHg	61.22 (25.94)	63.50 (25.63)	57.88 (26.05)	<0.001
BMI, kg/m2	32.76 (7.56)	33.08 (7.86)	32.29 (7.07)	0.087
Waist circumference, cm	110.89 (16.75)	110.94 (17.56)	110.81 (15.49)	0.887
HbA1c, %	7.32 (1.76)	7.20 (1.78)	7.50 (1.71)	0.003
UACR, mg/g	150.18 (702.79)	93.24 (497.73)	233.45 (918.06)	<0.001
eGFR, mL/min/1.73m²	81.17 (28.42)	86.28 (27.98)	73.68 (27.40)	<0.001
Triglyceride , mmol/L	1.86 (1.95)	1.90 (2.21)	1.82 (1.50)	0.453
Total cholesterol , mmol/L	4.61 (1.16)	4.73 (1.19)	4.45 (1.09)	<0.001
HDL‐C, mmol/L	1.24 (0.35)	1.24 (0.33)	1.25 (0.37)	0.369
LDL‐C, mmol/L	2.53 (0.94)	2.63 (0.93)	2.38 (0.93)	**<0.001**
FPG, mmol/L	8.71 (3.50)	8.56 (3.40)	8.93 (3.62)	0.105
SII	588.21 (374.16)	562.41 (323.43)	625.94 (435.32)	0.004
SIRI	1.43 (1.10)	1.34 (0.94)	1.57 (1.29)	<0.001

Note: Statistical significance was considered at p < 0.05, and results were marked in bold.

Abbreviations: BMI, body mass index; DBP, diastolic blood pressure; DM, diabetes mellitus; DR, diabetic retinopathy; eGFR, estimated glomerular filtration rate; FPG, fasting plasma glucose; HbA1c, glycosylated hemoglobin A1c; HDL‐C, high‐density lipoprotein cholesterol; LDL‐C, low‐density lipoprotein cholesterol; NDR, nondiabetic retinopathy; SBP, systolic blood pressure; SII, systemic immune‐inflammation index; SIRI, systemic immune‐inflammation response index; UACR, urinary albumin‐to‐creatinine ratio.

^a^Mean ± SD for continuous variables: p value was calculated by the weighted linear regression model; (%) for categorical variables: p value was calculated by the weighted *χ*2 test.

The baseline characteristics of the participants, categorized by DM duration groups, are shown in Table 1. Compared with participants in group 1 (DM duration ≤ 10 years), participants in group 2 (DM duration > 10 years) exhibited several notable differences. Specifically, group 2 participants were older, had a higher incidence of DR, a higher prevalence of anemia, and a greater proportion using insulin. Additionally, they had a higher percentage of individuals with less than a high school education and a greater prevalence of hypertension and DN. Furthermore, participants in group 2 had elevated levels of HbA1c and UACR, as well as higher SII and SIRI indices. However, they were diagnosed with diabetes at a younger age, had lower DBP, lower eGFR, and lower levels of TC and LDL‐C (all *p* < 0.05).

### 3.2. Association Between Risk Factors and DR Across Different DM Duration Groups

Univariate logistic regression analyses were performed to explore potential risk factors for DR across different DM duration groups. The results, detailed in Table 2, indicated that insulin use and DN were positively associated with DR in both group 1 and group 2. In addition, education level, specifically having a college degree or higher, was negatively associated with DR in both groups. For group 1, the following factors were positively correlated with DR: other races, HbA1c, TC, LDL‐C, and FPG. Conversely, earlier age of diabetes diagnosis and higher family income‐to‐poverty ratio were negatively associated with DR. In group 2, anemia and UACR were positively associated with DR, whereas eGFR was negatively associated with DR (all *p* < 0.05). The correlation analysis of potential factors related to the occurrence of DR is shown in Figure S1.

**Table 2:** Factors associated with diabetic retinopathy by diabetes duration groups: Univariable survey‐weighted logistic regression analyses.

Variable		≤ 10 years		>10 years	
		OR(95%C)	*p*	OR(95%CI)	*p*
Age in years at screening (per 1 year increase)		0.99(0.98 ‐ 1.00)	0.127	1.01(0.99 ‐ 1.03)	0.247
Sex: Male=1, female=2 (reference: male)	1	Ref.		Ref.	
	2	0.82(0.54 ‐ 1.25)	0.359	1.01(0.65 ‐ 1.58)	0.959
Age of DM diagnosis (per 1 year increase)		0.98(0.97 ‐ 1.00)	0.018	0.99(0.98 ‐ 1.00)	0.161
Anemia: NO=0, YES=1 (reference: NO)	0	Ref.			
	1	0.67(0.34 ‐ 1.30)	0.232	1.97(1.22 ‐ 3.19)	0.006
Use of insulin: Nonuse=0, current use=1 (reference: nonuse)	0	Ref.		Ref.	
	1	2.91(1.70 ‐ 4.98)	<0.001	2.97(1.98 ‐ 4.44)	<0.001
Race: Mexican American=1, other Hispanic=2, non‐Hispanic White=3, non‐Hispanic Black=4, other races ‐ including multiracial =5 (reference: Mexican American)	1	Ref.			
	2	1.96(1.00 ‐ 3.85)	0.051	1.16(0.59 ‐ 2.30)	0.660
	3	0.82(0.43 ‐ 1.59)	0.560	1.06(0.60 ‐ 1.85)	0.842
	4	1.25(0.67 ‐ 2.33)	0.486	1.36(0.73 ‐ 2.52)	0.326
	5	2.41(1.18 ‐ 4.89)	0.016	1.48(0.68 ‐ 3.23)	0.325
Education: Less than high school=1, high school or equivalent=2, college or above=3 (reference: less than high school)	1	Ref.		Ref.	
	2	0.63(0.33 ‐ 1.19)	0.151	0.82(0.47 ‐ 1.42)	0.471
	3	0.48(0.29 ‐ 0.81)	0.006	0.50(0.30 ‐ 0.83)	0.008
Family income‐poverty ratio level: <1.5=1, 1.5‐3.5=2, ≥3.5=3 (reference: <1.5)	1	Ref.		Ref.	
	2	0.44(0.25 ‐ 0.77)	0.005	0.88(0.53 ‐ 1.47)	0.621
	3	0.30(0.15 ‐ 0.60)	<0.001	0.64(0.36 ‐ 1.15)	0.137
Smoking status: Never =1, former=2, current=3 (reference: never)	1	Ref.		Ref.	
	2	0.65(0.41 ‐ 1.04)	0.069	0.72(0.46 ‐ 1.12)	0.140
	3	0.98(0.55 ‐ 1.77)	0.952	0.66(0.35 ‐ 1.24)	0.190
Alcohol intake: Nondrinkers = 0, drinkers = 1 (reference: nondrinkers)	0	Ref.		Ref.	
	1	0.67(0.40 ~ 1.14)	0.137	0.67(0.40 ~ 1.10)	0.112
Hypertension: NO=0, YES=1 (reference: NO)	0	Ref.		Ref.	
	1	1.36(0.80 ~ 2.30)	0.249	1.00(0.56 ~ 1.81)	0.992
Hyperlipidemia: NO=0, YES=1 (reference: NO)	0	Ref.		Ref.	
	1	1.06(0.57 ~ 1.97)	0.845	0.86(0.54 ~ 1.39)	0.538
Diabetic nephropathy: NO=0, YES=1 (reference: NO)	0	Ref.		Ref.	
	1	1.81(1.16 ~ 2.84)	0.010	2.31(1.48 ~ 3.61)	<0.001
SBP (per 1 mmHg increase)		1.00(1.00 ~ 1.01)	0.910	1.00(1.00 ~ 1.01)	0.499
DBP (per 1 mmHg increase)		1.00(0.99 ~ 1.01)	0.675	1.00(0.99 ~ 1.01)	0.952
BMI (per 1 kg/m2 increase)		0.99(0.97 ~ 1.02)	0.466	1.00(0.97 ~ 1.03)	0.911
Waist circumference (per 1 cm increase)		1.00(0.99 ~ 1.01)	0.622	1.00(0.99 ~ 1.01)	0.884
HbA1c (per 1% increase)		1.20(1.09 ~ 1.34)	<0.001	1.05(0.93 ~ 1.20)	0.414
UACR (per 1 mg/g increase)		1.00(1.00 ~ 1.00)	0.187	1.00(1.00 ~ 1.00)	0.033
eGFR (per 1 mL/min/1.73m² increase)		1.00(1.00 ~ 1.01)	0.304	0.98(0.98 ~ 0.99)	<0.001
Triglyceride (per 1 mmol/L increase)		1.05(0.97 ~ 1.13)	0.238	0.92(0.79 ~ 1.08)	0.327
Total Cholesterol (per 1 mmol/L increase)		1.28(1.08 ~ 1.52)	0.005	0.96(0.77 ~ 1.20)	0.730
HDL‐C (per 1 mmol/L increase)		1.27(0.74 ~ 2.19)	0.385	1.05(0.61 ~ 1.82)	0.848
LDL‐C (per 1 mmol/L increase)		1.31(1.04 ~ 1.66)	0.023	1.01(0.77 ~ 1.32)	0.949
FPG(per 1 mmol/L increase)		1.09 (1.03 ~ 1.16)	0.003	1.02(0.95 ~ 1.08)	0.647
SII (per 1 increase)		1.00(1.00 ~ 1.00)	0.436	1.00(1.00 ~ 1.00)	0.681
SIRI (per 1 increase)		1.00(0.82 ~ 1.22)	0.978	0.99(0.86 ~ 1.12)	0.831

Note: Statistical significance was considered at p < 0.05, and results were marked in bold.

Abbreviations: BMI, body mass index; CI, confidence interval; DBP, diastolic blood pressure; DM, diabetes mellitus; DR, diabetic retinopathy; eGFR, estimated glomerular filtration rate; FPG, fasting plasma glucose; HbA1c, glycosylated hemoglobin A1c; HDL‐C, high‐density lipoprotein cholesterol; LDL‐C, low‐density lipoprotein cholesterol; OR, odds ratio; SBP, systolic blood pressure; SII, systemic immune‐inflammation index; SIRI, systemic immune‐inflammation response index; UACR, urinary albumin‐to‐creatinine ratio.

### 3.3. Separate and Joint Effects of DM Duration and Anemia on the Risk of DR

The analysis of the relationship between DR occurrence, DM duration, and anemia status, as shown in Table 3, yielded the following results:

**Table 3:** Separate and joint effects of anemia and diabetes duration on the risk of diabetic retinopathy.

	Unadjusted		Adjusted					
			Model 1		Model 2		Model 3	
	OR (95% CI)	*p*	OR (95% CI)	*p*	OR (95% CI)	*p*	OR (95% CI)	*p*
Separate effects								
DM duration group								
≤ 10 years	Ref.		Ref.		Ref.		Ref.	
> 10 years	2.07 (1.47–2.92)	**< 0.001**	2.24 (1.56–3.23)	**< 0.001**	1.53 (1.08–2.18)	**0.018**	1.53 (1.08–2.16)	**0.017**
Anemia status								
Nonanemia	Ref.		Ref.		Ref.		Ref.	
Anemia	1.57 (1.13–2.18)	**0.007**	1.43 (1.01–2.02)	**0.043**	1.21 (0.83–1.77)	0.327	1.22 (0.83–1.80)	0.313
Joint effects								
Nonanemia								
DM duration ≤ 10 years	Ref.		Ref.		Ref.		Ref.	
DM duration > 10 years	1.75 (1.17–2.59)	**0.006**	1.91 (1.26–2.89)	**0.002**	1.29 (0.88–1.90)	0.191	1.29 (0.88–1.88)	0.188
Anemia								
DM duration ≤ 10 years	0.67 (0.34–1.29)	0.228	0.63 (0.32–1.23)	0.173	0.54 (0.27–1.08)	0.082	0.54 (0.27–1.10)	0.090
DM duration > 10 years	3.45 (2.22–5.34)	**< 0.001**	3.51 (2.19–5.63)	**< 0.001**	2.21 (1.29–3.78)	**0.004**	2.24 (1.30–3.84)	**0.004**

*Note:* Statistical significance was considered at p < 0.05, and results were marked in bold. Model 1: adjusted for survey year, age, sex, and race; Model 2: adjusted for survey year, age, sex, race, use of insulin, educational level, and diabetic nephropathy; Model 3: adjusted for survey year, age, sex, race, use of insulin, educational level, diabetic nephropathy, family income‐poverty ratio level, hemoglobin A1c, urinary albumin: creatinine ratio, estimated glomerular filtration rate, and total cholesterol.

Abbreviations: CI, confidence interval; DM, diabetes mellitus; OR, odds ratio.

In the unadjusted model, participants with DM duration > 10 years had a significantly higher risk of DR compared with those with DM duration ≤ 10 years (*OR* = 2.07, 95% CI 1.47–2.92, *p* < 0.001). Similarly, participants with anemia exhibited a significantly higher risk of DR compared with non‐anemic participants (*OR* = 1.57, 95% CI 1.13–2.18, *p* = 0.007). After sequential adjustment for covariates across different models (Models 1–3), a DM duration of > 10 years remained an independent risk factor for DR (Model 3: *OR* = 1.53, 95% CI 1.08–2.16, *p* = 0.017). In contrast, the association between anemia and DR lost statistical significance after controlling for potential confounders (Model 3: *OR* = 1.22, 95% CI 0.83–1.80, *p* = 0.313).

Further analysis of their joint effects revealed that, compared with the reference group (DM duration ≤ 10 years and no anemia), participants with both DM duration > 10 years and anemia had a significantly higher risk of DR. In the unadjusted model, the OR was 3.45 (95% CI 2.22–5.34, *p* < 0.001). This elevated risk remained significant even after full adjustment for confounders (Model 3: *OR* = 2.24, 95% CI 1.30–3.84, *p* = 0.004).

## 4. Discussion

Our findings indicate that insulin use, DN, and education level are significantly associated with the occurrence of DR, regardless of DM duration. A longer duration of DM (> 10 years) is an independent risk factor for DR, whereas anemia alone does not show a significant independent association with DR after adjusting for relevant potential confounders. However, the coexistence of prolonged DM duration and anemia appears to have a synergistic effect, significantly increasing the risk of DR.

We found that insulin use was significantly associated with the occurrence of DR, regardless of DM duration. It is well‐known that insulin therapy for DM is typically initiated in patients with pre‐existing poor glycemic control, which may reflect more severe or advanced stages of diabetes. As such, the observed association between insulin use and DR could be influenced by confounding by indication, as patients requiring insulin may have more uncontrolled diabetes or additional comorbidities that independently increase the risk of DR. Furthermore, insulin use itself may contribute to the risk of DR through mechanisms such as the larger and more rapid fluctuations in blood glucose levels [27], which have been found to be independent risk factors for diabetic complications in previous studies [28]. Additionally, the use of insulin can result in the generation of oxidative stress through the formation of reactive oxygen species, which in turn can lead to the overexpression of vascular endothelial growth factor (VEGF) [27, 29]. Recent studies have also shown that the impact of insulin on the retina could be reflected in retinal microvascular imaging parameters, increasing the risk of DR and cardiovascular events in patients with T2DM [30]. Although these findings underscore the importance of closely monitoring insulin‐treated patients for DR, we acknowledge that insulin use may be a marker of disease severity rather than a direct causal factor. Therefore, routine fundus examinations should be conducted for insulin‐treated T2DM patients to facilitate early DR detection while considering the underlying severity of their diabetes.

DN has been identified as a significant risk factor for DR. Several studies have highlighted the potential link between these two complications [31–33]. Prolonged hyperglycemia contributes to the accumulation of oxidative stress, activation of inflammatory pathways, and dysfunction of microvascular endothelial cells, all of which are key pathological processes common to both DN and DR [31, 34]. The presence of DN often correlates with more severe stages of DR [35]. Therefore, given the shared pathophysiological mechanisms, DN and DR may occur simultaneously or sequentially in individuals with diabetes. The close relationship between these two complications underscores the importance of early screening and management to prevent their progression and reduce the risk of severe outcomes.

Higher education levels in participants with DM are negatively associated with the occurrence of DR. Lower levels of education are often associated with “low attendance” in DR screening [36, 37]. Participation in DR screening is influenced by long‐term adherence to ocular health care. Additionally, individuals with lower levels of education may have a reduced ability to internalize health information and translate it into lifestyle changes [38, 39]. This could explain why the highest level of education has a protective effect against the presence of DR. Therefore, it is essential to prioritize education and outreach for individuals with lower levels of education. Tailored educational programs that focus on the importance of regular screening, self‐management, and lifestyle changes can help improve their adherence to ocular health care and reduce the risk of DR in this population.

A prolonged DM duration is well‐established as a significant risk factor for DR [4, 5, 40]. According to a study from Debre Markos, patients with a diabetes duration exceeding 10 years have a significantly increased risk of DR, with the risk nearly quadrupling compared with those with a shorter diabetes duration [41]. This finding aligns with the results of our study, where we observed a 1.53‐fold increased risk of DR in individuals with DM duration > 10 years. Prolonged exposure to high blood glucose levels induces endothelial dysfunction, increased oxidative stress, and changes in vascular permeability, all of which contribute to retinal damage [42, 43]. Although anemia did not show a significant independent association with DR after adjusting for confounders, the presence of anemia in individuals with long‐duration DM appears to exacerbate the risk of DR. Earlier population‐based research has identified anemia as an independent risk factor for DR [8, 14, 15]. Significant reductions in hemoglobin, hematocrit, and RBC levels have been reported by Traveset et al. [14] in patients with severe DR. It is believed that a reduction in hemoglobin concentration can impair the blood′s capacity to transport oxygen, resulting in hypoxia, which plays a crucial role in the progression of DR [44]. In response to hypoxia, retinal endothelial cells, pericytes, and pigment epithelial cells express VEGF, which stimulates angiogenesis, leading to neovascularization and increasing capillary permeability, resulting in retinal edema [45]. In addition, severe anemia can elicit compensatory physiological responses to tissue hypoxia, including increases in cardiac output and systemic blood flow [46]. However, in the diabetic state, impaired vascular autoregulation and reduced microvascular resilience may limit the ability of the retinal circulation to adapt to these hemodynamic changes [47]. Consequently, the retinal microvasculature may be exposed to heightened shear stress under high‐flow conditions, potentially exacerbating endothelial injury, increasing vascular permeability, and accelerating microvascular damage [48].

Our analysis demonstrates a significant synergistic effect when both prolonged DM duration and anemia are present. In patients with both conditions, the risk of DR is substantially higher compared with those with either condition alone. This synergistic effect may be due to the combined vascular and metabolic stress imposed by both long‐term hyperglycemia and the compromised oxygen‐carrying capacity associated with anemia [14, 42]. For clinical management, addressing anemia in patients with long‐duration DM may potentially reduce its contributory effect on the occurrence of DR. Managing anemia, through interventions such as iron supplementation or other appropriate therapies, could improve tissue oxygenation and reduce retinal hypoxia, which may help in reducing the risk of DR [49].

To the best of our knowledge, this study is the first to analyze the effects of sociodemographic and systemic factors on DR according to stratification of DM duration. We included a large sample of individuals to provide sufficient statistical power. However, several limitations should be acknowledged. First, this was a cross‐sectional study, and more prospective studies will be needed in the future to explain the causal relationship between the factors studied and the presence of DR. Second, DR was defined solely based on self‐reported questionnaire data, which may lead to underreporting or misclassification of DR cases, especially during the early or asymptomatic stages. Third, the study was conducted in diabetic individuals in the United States. Whether these findings apply to other populations will need to be investigated further.

## 5. Conclusion

The sociodemographic and systemic risk factors for DR varied with DM duration. Insulin use, DN, and education level were significantly associated with DR across all DM duration groups. Notably, the combination of prolonged DM duration and anemia had a synergistic effect, significantly increasing the risk of DR.

## Author Contributions

**Yuan Liu:** conceptualization, methodology, software, project administration, writing—original draft. **Yang Meng:** conceptualization, methodology, software, project administration, writing—original draft. **Runping Duan:** resources, writing—review & editing. **Baoyi Liu:** resources, writing—review & editing. **Yuan Ma:** resources, writing—review & editing. **Chin-Ling Tsai:** writing—review & editing. **Ziye Chen:** writing—review & editing. **Zhuojun Xu:** writing—review & editing. **Zitong Chen:** writing—review & editing. **Guanli Zhu:** writing—review & editing. **Tao Li:** conceptualization, resources, writing—review & editing, supervision, funding acquisition. Yuan Liu and Yang Meng contributed equally to this work as co‐first authors.

## Funding

This study was supported by the National Natural Science Foundation of China (10.13039/501100001809; 82271093 and 82070972); Key Science and Technology Project of Guangzhou (202103000045); Guangdong Basic Research Center of Excellence for Major Blinding Eye Diseases Prevention and Treatment (2024‐PIZC‐010 and 2024‐RCPY‐009); and the Research Funds of the State Key Laboratory of Ophthalmology (2025QNMY09).

## Ethics Statement

This study involves human participants, and the National Center for Health Statistics Research Ethics Review Board approved all NHANES protocols.

## Consent

Participants gave informed consent to participate in the study before taking part.

## Conflicts of Interest

The authors declare no conflicts of interest.

## Supporting Information

Additional supporting information can be found online in the Supporting Information section.

## Supporting information


**Supporting Information 1** Table S1: List of variables with missing values and their corresponding percentages. LDL‐C, low‐density lipoprotein cholesterol; HDL‐C, high‐density lipoprotein cholesterol; SII, systemic immune‐inflammation index; SIRI, systemic immune‐inflammation response index; BMI, body mass index.

**Supporting Information 2** Figure S1: Correlation plot of Sociodemographic and systemic factors, stratified by diabetic retinopathy status. DM, diabetes mellitus; DR, diabetic retinopathy; NDR, nondiabetic retinopathy; HbA1c, hemoglobin A1c; UACR, urinary albumin‐to‐creatinine ratio; eGFR, estimated glomerular filtration rate; LDL‐C, low‐density lipoprotein cholesterol; FPG, fasting plasma glucose. Race 1: Mexican American, Race 2: Other Hispanic, Race 3: Non‐Hispanic White, Race 4: Non‐Hispanic Black, Race 5: Other races—Including multiracial. Pearson correlation coefficients are shown, with significance levels indicated by stars (*p* < 0.05:  ^∗^, *p* < 0.01:  ^∗∗^, and *p* < 0.001:  ^∗∗∗^).

## Data Availability

The data used in this study are available from the NHANES database at https://wwwn.cdc.gov/nchs/nhanes/Search/default.aspx.
