# Supplementary material for: Risk Factors for Diabetic Retinopathy Change With Diabetes Duration: Synergistic Effect of Long Duration and Anemia
Source: J Diabetes Res. 2026 May 5;2026:8611325. doi: 10.1155/jdr/8611325 (PMC13144550; doi:10.1155/jdr/8611325)
Supplement: Supplementary file 1 — Supporting Information 1 Table S1: List of variables with missing values and their corresponding percentages. LDL‐C, low‐density lipoprotein cholesterol; HDL‐C, high‐density lipoprotein cholesterol; SII, systemic immune‐inflammation index; SIRI, systemic immune‐inflammation response index; BMI, body mass index. [file JDR-2026-8611325-s001.docx]

**Supplementary Table.** List of Variables with Missing Values and Their Corresponding Percentages.

| Variable | Missing rate, % |
| --- | --- |
| Family income-poverty ratio level | 11.60 |
| Alcohol intake | 10.80 |
| LDL-c | 10.50 |
| Waist circumference | 7.76 |
| Triglyceride | 7.68 |
| HDL-c | 6.76 |
| Total Cholesterol | 6.76 |
| SII | 4.42 |
| SIRI | 4.42 |
| Anemia status | 4.26 |
| BMI | 2.90 |
| Hyperlipidemia | 2.61 |
| Educational level | 0.89 |
| Smoking status | 0.76 |
| Hypertension | 0.28 |
| Use of insulin | 0.04 |

LDL-C, Low-Density Lipoprotein Cholesterol; HDL-C, High-Density Lipoprotein Cholesterol; SII, Systemic Immune-Inflammation Index; SIRI, Systemic Immune-Inflammation Response Index; BMI, Body Mass Index.
