# Supplementary figures and images for: Risk Factors for Diabetic Retinopathy Change With Diabetes Duration: Synergistic Effect of Long Duration and Anemia
Source: J Diabetes Res. 2026 May 5;2026:8611325. doi: 10.1155/jdr/8611325 (PMC13144550; doi:10.1155/jdr/8611325)

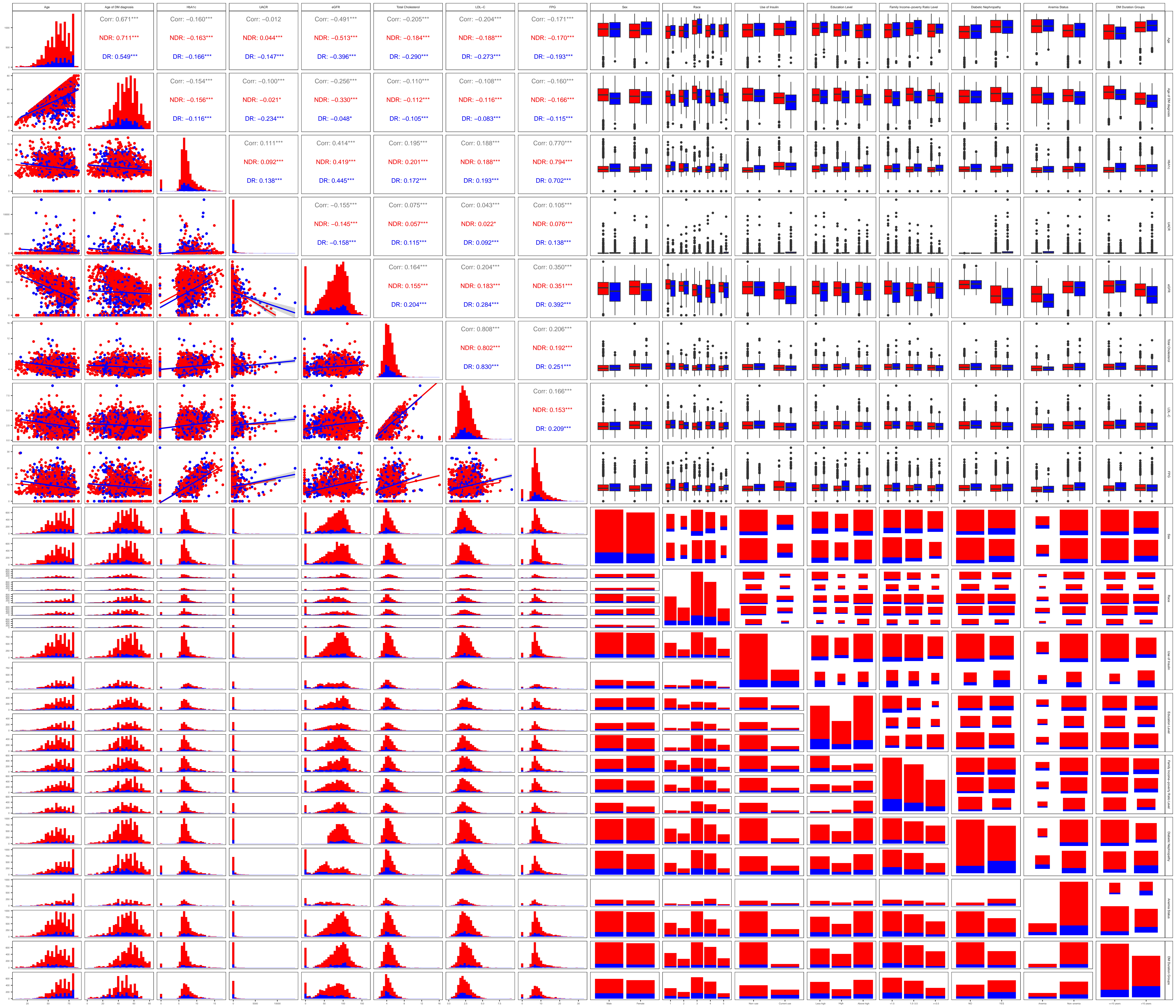

Supplement: Supplementary file 2 — Supporting Information 2 Figure S1: Correlation plot of Sociodemographic and systemic factors, stratified by diabetic retinopathy status. DM, diabetes mellitus; DR, diabetic retinopathy; NDR, nondiabetic retinopathy; HbA1c, hemoglobin A1c; UACR, urinary albumin‐to‐creatinine ratio; eGFR, estimated glomerular filtration rate; LDL‐C, low‐density lipoprotein cholesterol; FPG, fasting plasma glucose. Race 1: Mexican American, Race 2: Other Hispanic, Race 3: Non‐Hispanic White, Race 4: Non‐Hispanic Black, Race 5: Other races—Including multiracial. Pearson correlation coefficients are shown, with significance levels indicated by stars (p < 0.05: ∗, p < 0.01: ∗∗, and p < 0.001: ∗∗∗). [file JDR-2026-8611325-s002.pdf]
